# What’s in a Name? Michael N. Marsh, BTh, DPhil, DM, DSc, FRCP*

**DOI:** 10.5146/tjpath.2021.01553

**Published:** 2021-09-15

**Authors:** Arzu Ensari

**Affiliations:** Ankara University School of Medicine. Department of Pathology, Ankara, Turkey

Great minds deserve to be acknowledged while still alive though their true recognition usually takes place afterwards. I would like to believe that this was not the case for **Marsh** as for over fifty years, the name “**Marsh**” has dominated not only the world of coeliac disease but also the field of pathology, particularly, intestinal mucosal immunopathology with morphometric measurements and interpretation of normal vs pathological mucosae. It is my honourable duty to share the inspiring life story of a great scientific mind with my fellow pathologists and the future pathologists so that they appreciate, “**Marsh**” is more than a name never to be forgotten.

Michael Newton **Marsh** was born in Bristol, UK, of working-class parents. His education started at a local Grammar School and continued at the University of Leeds, School of Medicine. After his pre-clinical studies, he studied for a B.Sc. in Human Anatomy and Cell Biology which formed the basis of his future work on intestinal mucosal morphology and immunopathology. He then studied in Magdalen College, Oxford University and Oxford Clinical School and graduated BM, B.Ch. in 1962. After becoming a Member of the Royal College of Physicians, **Marsh** became Chief Resident at London’s Hammersmith Hospital where he met Sir Christopher Booth who was working on coeliac mucosae with the dissecting microscope. Inspired by his work, **Marsh** looked at mucosal biopsies with the “new” scanning electron microscope allowing him to discover the realm of intestinal villus and published this work in prestigious journals like the Lancet and the Gut in 1968.

**Marsh** got his MD in 1972 in Oxford and with a Medical Research Council Travelling Fellowship, he went to Boston Mass to start intestinal research using epon-embedded tissues cut at 1µm, stained with toluidine blue, and examined under oil-immersion optics which became central to his research activities on his return to England in early seventies. Between 1974 and 2000 he had tenure of an academic post in the Medical Faculty, University of Manchester, Hope Hospital. There he had his own histopathology lab next to his tiny office literary filled up by the image analyser he used for his morphometric studies which he published during the eighties under the common title of “**Morphometric analysis of small intestinal mucosa. I to IV” **numbered in roman style which he always preferred over Arabic numerals. It was the magical **“number III”** which introduced this great mind to a young pathology resident far away from where the paper was written I, as a 2nd year-pathology resident at the Department of Pathology, Ankara University School of Medicine presented this paper in one of the journal clubs in the department. The terms “image analysis” and “morphometry” caught my attention together with the name **Marsh** who very kindly responded to my letter written on a typewriter and the next year I was in his tiny office at Hope Hospital, University of Manchester for a brief period of three months. Time was short but achievement was grand on my account as **Marsh** offered a PhD upon completion of my residency in pathology.

**Marsh** strongly believed that the lectures in the Sir William Dunn School of Pathology introduced him to the mucosal lymphocyte which later became one of his favourite cell types in his research, in short, the “IEL (intraepithelial lymphocyte)”. Besides his morphometry papers he published another series which were gathered under the common title of **“Studies of intestinal lymphoid tissue”** again enumerated by roman numbers of I to XV mainly focusing on mucosal inflammatory cell composition in coeliac disease and related enteropathies. However, he was always curious why IEL numbers were determined against 100 epithelial cells and similarly critical about the use of high-power fields to determine comparative cell numbers. **Marsh** came up with a solution by using a 100x100µm test square of muscularis mucosae upon which to create the 3-dimensional features of each specimen, by compute to produce accurate, absolute measurements of any mucosa. This achieved a far greater series of comparative mucosal observations that provide the panoramic view of the mucosal pathology spectrum that took him to construct 20 years and publish the triumph of his life’s work in Gastroenterology in 1992** – the realisation that mucosal responses to gluten in susceptible subjects reveal progressive time-dependent responses from “normal” to flat, and also that pathological staging is independent of symptomatology. The four phases of his mucosal spectrum originally designated “normal; infiltrative; infiltrative-hyperplastic; and flat”, to his great surprise, came to be known eponymously as **Marsh** 0, I, II, III, or the **Marsh** Classification. This ground-breaking article has received over 2,050 international citations which possibly is a record for any author but also for the journal, too!

**Figure:**
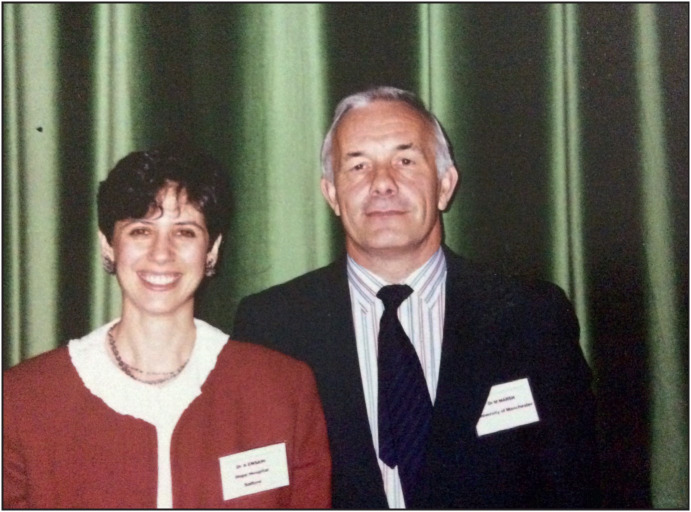
BSG Meeting in Warwick, 1992. Arzu Ensari, Michael N. Marsh

From a pathologist’s view, “**Marsh** classification” should not be regarded merely as a classification but a scheme to understand mucosal pathology of coeliac disease. I, wholeheartedly believe that it has made the most powerful impact on the interpretation of small-intestinal biopsies by pathologists. Despite being a physician, his classification of mucosal “pathology” not only improved our understanding of the biology of the disease but also enabled the recognition of cases with increased IEL counts in an otherwise normal mucosa which was later defined as “microscopic enteritis” by **Marsh** and a group of distinguished colleagues. As our understanding of mucosal immunity has developed over the years, it became clear that there was indeed no alternative to the validity of **Marsh** scheme as reflective of the innate and adaptive phases of the mucosal T-lymphocyte response to luminal antigen.

**Marsh** has always objected to the term “atrophy”, and accompanying designations “partial”, “subtotal”, “total” villous destruction, and the subdivisions **Marsh** IIIa, b, c to define villous architectural changes. His concept was based on the dynamic studies which showed that the mucosa did not at all undergo a process of atrophy, but it rather demonstrated a hyperplastic state characterized by elongation of the crypts and widening of the lamina propria by inflammation. This observation is also supported by the finding that the height of the mucosa is not changed when an infiltrative lesion progresses to a flat lesion. Nevertheless, these subdivisions continue to appear in most papers on coeliac mucosa without any thought of precise (measured) definition. Moreover, we know of no paper in which these subdivisions were ever employed to sharpen diagnosis, inform treatment, or anticipate future prognostic effects of dietary control.

The occurrence of functional plasma cells in the large bowel greatly excited Marsh, which one of his students (AE, with Dr Duncan Loft) would later be asked to pursue in the diagnosis of coeliac disease following rectal gluten challenge. Marsh was disappointed both by the lack of interest in this simple approach to diagnosis, since many still believed coeliac disease only to involve the small intestine, as well as by the apparent lack of understanding of the mesenteric immune system and how it could be mobilised for clinical purposes. This, too, proves his visionary approach since the “full house pattern”of mucosal inflammation including lymphocytic oesophagitis, duodenitis and colitis in coeliac disease is now a widely accepted concept.

**Marsh** has always complained of the weakness of light microscopic examination which allows two-dimensional evaluation whereas to him it was crucial to understand the complexity of the three-dimensional features of small intestinal morphology. He moved further to collaborate with groups working on high-tech systems including gene expression profiling methods to highlight the need of transition “from 2-dimensional to 3-dimensional approach”. Unfortunately, a deadly disease with no mercy, wouldn’t allow him to continue his pursuit of truth…

This is a very brief summary of the life of a great mind devoted to a high level of rigorous scientific investigation in discovering novel approaches to elucidating intestinal morphology dominated by the Classification. He gave over 100 lectures worldwide, published 200+ papers, and three scientific books: Immunopathology of the Small Intestine (Wiley 1987); Coeliac Disease (Blackwell Scientific 1992); Methods in Molecular Medicine (Humana Press 2000).

On a personal note, however, my “Dr. **Marsh”**, who later became “Michael (upon his request)” was more than a name: an accomplished musician playing the organ, Bach being his favourite composer, a Theology scholar with a degree from Oxford, a wizard of English language (one may need a dictionary when reading his papers), a man with very sharp intellect, typical British humour, and incredible honesty which did not help him climb the stairs with less effort nor let him become member of any particular group. As for today, **Marsh** himself had become an internationally recognised “name” not only to the scientific coeliac disease innovative research, but also for global practicing pathologists dealing with small intestinal biopsies. The name is **Marsh**…

## Footnotes

** *Marsh M (1992). “Gluten, major histocompatibility complex, and the small intestine. A molecular and immunobiologic approach to the spectrum of gluten sensitivity (‘coeliac sprue’)”. Gastroenterology 102 (1): 330–54.*

